# Effects of plyometric training on muscle–tendon mechanical properties and behavior of fascicles during jumping

**DOI:** 10.14814/phy2.15073

**Published:** 2021-10-29

**Authors:** Keitaro Kubo, Toshihiro Ikebukuro, Hideaki Yata

**Affiliations:** ^1^ Department of Life Science The University of Tokyo Meguro, Tokyo Japan; ^2^ Sports Science Laboratory Wako University Machida, Tokyo Japan

**Keywords:** fascicle, hysteresis, plantar flexion, stiffness, ultrasonography

## Abstract

The purpose of this study was to investigate the effects of plyometric training on the muscle–tendon mechanical properties and behavior of fascicles during jumping in order to elucidate the mechanisms of improved jump performance due to plyometric training. Eleven subjects completed a 12‐week unilateral training program for plantar flexors. Active muscle stiffness with (100°·s^−1^) and without (250°·s^−1^) stretch reflex were calculated according to changes in muscle force and fascicle length during fast stretching after submaximal isometric contractions. Stiffness and hysteresis of tendon were measured using ultrasonography during ramp and ballistic contractions. Three kinds of unilateral jump heights using only ankle joint (no counter‐movement jump: no‐CMJ; counter‐movement jump: CMJ; drop jump: DJ) on sledge apparatus were measured. During jumping, electromyographic activities (mEMG) of plantar flexors and fascicle length of the medial gastrocnemius muscle were measured. Active muscle stiffness at 250 and 100°·s^−1^ and maximal tendon elongation during ballistic contraction significantly increased after training. Tendon hysteresis during ballistic contraction significantly decreased after training, whereas that during ramp contraction did not. The heights of three jump tests, the ratio of mEMG during eccentric to that during concentric phases for CMJ, and the amount of fascicle shortening and shortening velocity during eccentric phase of DJ significantly increased after training. These results suggest that an increase in CMJ height was associated with changes in the muscle–tendon mechanical properties and muscle activation strategy, whereas an increase in DJ height could be explained by changes in the muscle–tendon mechanical properties, but not muscle activation strategy.

## Graphical abstract

We demonstrated that tendon extensibility and active muscle stiffness increased and tendon hysteresis decreased after 12‐week plyometric training. Furthermore, the optimization of muscle‐tendon behavior and muscle activation strategy were observed during jumping due to training‐induced changes in the muscle‐tendon mechanical properties.

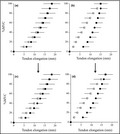

## 1 INTRODUCTION

In competitive sports, plyometric training is used to enhance jumping and sprinting abilities. The proposed explanations for these improvements are changes in the muscle activation strategies (Chimera et al., 2004; Hakkinen et al., 1990; Toumi et al., 2004) and the muscle–tendon mechanical properties (Foure et al., 2010; Kubo et al., 2017; Kubo, Morimoto, Komuro, Yata, et al., 2007). For the latter, several studies demonstrated that the mechanical properties of tendons (e.g., stiffness) are important for both muscle power production and efficiency during various exercises (Kawakami et al., 2002; Lichtwark & Wilson, 2007). To date, however, studies on the effects of plyometric training on tendon stiffness have been limited, and a consensus view has not been reached (Burgess et al., 2007; Foure et al., 2009, 2010; Hirayama et al., 2017; Kubo et al., 2017; Kubo, Morimoto, Komuro, Yata, et al., 2007; Wu et al., 2010). Furthermore, few studies have shown plyometric training‐induced changes in tendon hysteresis (Foure et al., 2010; Kubo et al., 2017), although tendon hysteresis affects the reuse of stored elastic energy during stretch‐shortening cycle exercises (Kubo et al., 2005; Wang et al., 2012). We previously reported that tendon hysteresis measured during ramp (lower strain rate) and ballistic (higher strain rate) contractions did not change after 12 weeks of plyometric training (Kubo et al., 2017). On the other hand, muscle contractions at a submaximal level are often repeated continuously during normal exercises (e.g., jogging), although tendon hysteresis has been evaluated during single contraction from resting to maximal exertion levels. Therefore, we should investigate the effect of plyometric training on tendon hysteresis measured during repeated submaximal contractions, which simulate normal exercise.

In addition to tendon properties as described above, we need to determine the effect of plyometric training on the mechanical properties of muscles under active conditions in order to reveal the mechanisms of plyometric training‐induced improvements of jumping and sprinting abilities. We previously invented a method to evaluate muscle stiffness under active conditions (i.e., active muscle stiffness) according to changes in the exerted torque and fascicle length during fast stretching (Kubo, 2014). Using this technique, we reported that active muscle stiffness significantly increased after 12 weeks of plyometric training (Kubo et al., 2017). However, active muscle stiffness obtained using this technique represented the intrinsic characteristic of muscle without any potential neural effects, for example, stretch reflex (Kubo, 2014). Recently, we proposed a method to evaluate active muscle stiffness including the effect of the stretch reflex according to changes in torque and fascicle length during a longer duration of slower stretching compared with the procedure reported by Kubo (2014) (Kubo et al., 2018). Previous studies demonstrated that performances during stretch‐shortening cycle exercises were affected by the stretch reflex (Horira et al., 1996; Kuitunen et al., 2002). Accordingly, a plyometric training‐induced increase in active muscle stiffness including the effect of the stretch reflex would be related to that in jump performance.

Over the last two decades, knowledge concerning the dynamics of muscles and tendons during stretch‐shortening cycle exercises has been accumulated (e.g., Fukunaga et al., 2001). However, few studies have tried to determine the training‐induced changes in muscle–tendon behavior during jumping and running (Hirayama et al., 2017; Werkhausen et al., 2018). Regarding the mechanisms of enhancement of stretch‐shortening cycle performance due to plyometric training, most previous studies just considered changes in muscle activities during exercises and muscle–tendon mechanical properties (e.g., Kubo et al., 2017). Therefore, we may be able to directly clarify the mechanisms of enhancement of stretch‐shortening cycle performance after plyometric training according to the training‐induced changes in muscle–tendon behavior during stretch‐shortening cycle exercises.

In this study, we determined the changes in the muscle–tendon mechanical properties and the behavior of fascicles during jumping in order to elucidate the mechanisms underlying improved jumping ability due to plyometric training. We hypothesized that tendon elongation during ballistic contraction and active muscle stiffness (especially that including the effect of the stretch reflex) increased and tendon hysteresis measured during repeated submaximal contractions decreased after plyometric training. In addition, the optimization of muscle–tendon behavior may be observed during jumping due to training‐induced changes in the muscle–tendon mechanical properties.

## 2 METHODS

### 2.1 Subjects

Twenty‐one healthy male college students volunteered to participate in the present study. Subjects were divided into to a training group (*n* = 11; age: 20.9 ± 1.6 years, height: 169.5 ± 5.0 cm, body mass: 62.5 ± 7.4 kg, mean ± SD) and control group (*n* = 10; age: 21.0 ± 1.4 years, height: 170.2 ± 5.9 cm, body mass: 63.0 ± 6.9 kg). They did not engaged in any regular exercise training for at least 2 years before this experiment. They were given a detailed explanation of the procedures as well as the purpose of this study. Written informed consent was obtained from all subjects. This study confirmed to the *Declaration of Helsinki* and was approved by the office of the Department of Sports Sciences, The University of Tokyo, and complied with their requirements for human experimentation.

### 2.2 Training

The training group completed unilateral (right side) plyometric training for 12 weeks (3 days/week). The training protocol involved two kinds of jump exercise (hopping and drop jumps [DJ]) using a sledge apparatus (VR‐4100; Cybex Corp.). The training protocol was the same as that used in our previous studies (Kubo et al., 2017; Kubo, Morimoto, Komuro, Yata, et al., 2007). In the hopping jump, subjects held the ankle in a maximal plantar flexed position in the beginning. Subsequently, subjects exerted plantar flexion torque to a maximal dorsiflexed position, and then rebounded to exert plantar flexion torque until the toe lifted off the footplate. In the DJ, subjects were dropped from a height of 20 cm from the surface of the footplate of this apparatus with the assistance of an experimenter. After landing on the footplate, they voluntarily stopped the falling movement by exerting eccentrically plantar flexion torque. Subsequently, they started plantar flexion and took off. Each exercise (hopping and DJs) was performed five sets with a 30‐s rest between each, which consisted of unilateral plantar flexion at 40% of one‐repetition maximum with 10 repetitions per set. Measurement of the one‐repetition maximum was conducted every 4 weeks to adjust the training load. At the end of the training session, the one‐repetition maximum significantly increased by 38.6 ± 14.1% (*p *< 0.001).

### 2.3 Muscle thickness and tendon cross‐sectional area

The thickness of the medial gastrocnemius muscle (MG), lateral gastrocnemius muscle (LG), and soleus muscle (SOL) was measured with an ultrasonic apparatus (SSD‐900; Aloka), using the procedure described by Kubo et al. (2014). Cross‐sectional ultrasonic images were obtained at proximal levels of 30% (MG and LG) and 50% (SOL) of the lower leg length, from the popliteal crease to the center of the lateral malleolus. These measured sites for the muscle thickness were the same as those described in our previous studies (Kubo et al., 2014, 2017). In addition, we also measured the cross‐sectional area of the Achilles tendon at the height of the lateral malleolus using an ultrasonic apparatus. The repeatability of measurements of the muscle thickness and tendon cross‐sectional area was confirmed in our previous study (Kubo et al., 2014).

### 2.4 Active muscle stiffness

Subjects lay prone on a test bench, and the waist and shoulders were secured by adjustable lap belts and held in position. The ankle joint was set at 100° (with the foot perpendicular to the tibia = 90° with angles more than 90° on plantar flexion) with the knee joint at full extension, and the foot tightly secured by two straps to the footplate of a custom‐designed dynamometer (AO‐K01; Applied Office), with angular velocities of up to around 400°·s^−1^ during relaxed condition and 250°·s^−1^ during isometric contractions. After a standardized warm‐up and submaximal contractions to habituate to the tests, subjects were asked to perform twice 3‐s maximal voluntary isometric contractions (MVC) for plantar flexion at a 100° ankle angle. The highest MVC value was used to determine the target torque during the measurement of active muscle stiffness.

**FIGURE 1:**
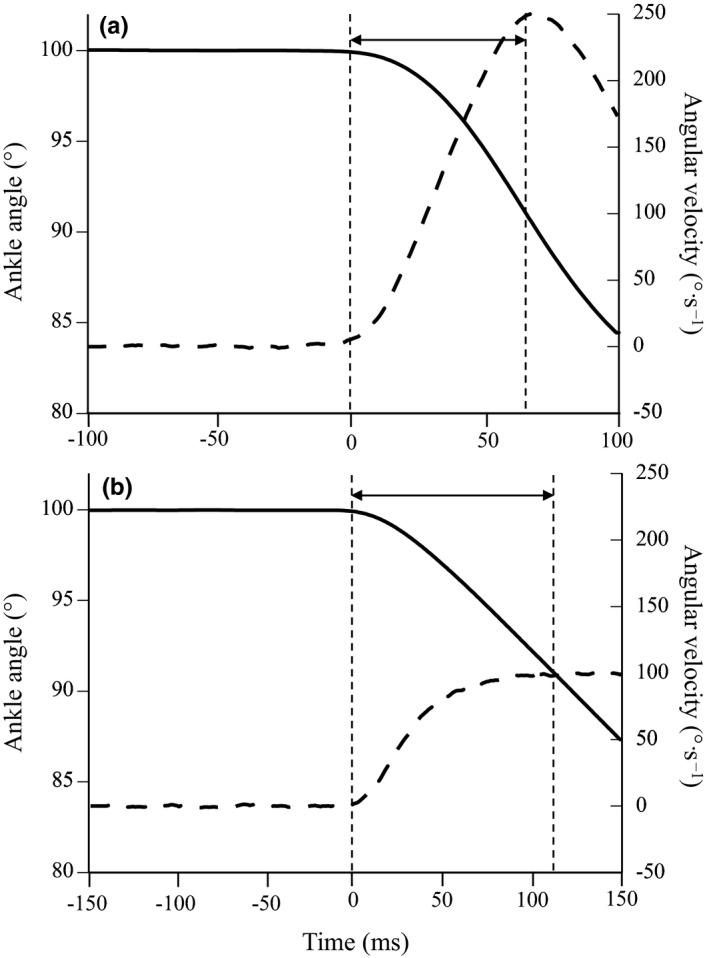
Typical examples of changes in the ankle angle (solid line) and angular velocity (dotted line) under 250°·s^−1^ (a) and 100°·s^−1^ (b) conditions. Active muscle stiffness was calculated from the range of double arrows

After a 5‐min rest period, measurements of active muscle stiffness were performed at two angular velocities (peak angular velocities were 250 and 100°·s^−1^, Figure 1) using a previously described procedure (Kubo & Ikebukuro, 2019; Kubo et al., 2018). The dynamometer was programmed to apply dorsiflexion from 100 to 80°. For the 250°·s^−1^ condition, we analyzed a 60‐ms period after the onset of stretch in order to avoid any potential neural intervention (Blanpied & Smidt, 1992; Kubo, 2014). For the 100°·s^−1^ condition, we analyzed a 110‐ms period after the onset of stretch in order to include any potential neural intervention, for example, stretch reflexes. During these analyzed periods (60 ms for 250°·s^−1^ and 110 ms for 100°·s^−1^), the range of motion was about 8° (Figure 1).

An additional measurement for each angular velocity was performed two times at 0% MVC. The averaged torque during the relaxed condition (caused by inertia and passive elasticity) was subtracted from the measured torque during each of the active stretch trials. The measurement of active muscle stiffness was performed two times per condition (250 and 100°·s^−1^) at three levels of submaximal torque (30%, 50%, and 70% MVC). The measured values given were the means of two tests. The muscle force (*F*_m_) was calculated from the ankle joint torque (TQ) measured by the dynamometer using the following equation (e.g., Kubo, 2014):

$$F_{m}=k\cdot \text{TQ}\cdot {\text{MA}}^{‐1},$$

where *k* is the relative contribution of the physiological cross‐sectional area of MG within the plantar flexor muscles (Fukunaga et al., 1996), and MA is the moment arm length of the triceps surae muscles with the ankle joint at 90° (Maganaris et al., 1998).

A real‐time ultrasonic apparatus (SSD‐6500; Aloka) was used to measure the fascicle length of MG during the measurement of active muscle stiffness. Ultrasonic images were captured at 100 Hz in the computer memory of the apparatus and were electrically synchronized them with other data (torque and joint angle) (e.g., Kubo, 2014). The slope of muscle force—fascicle length curve within the analyzed period analyzed was adopted as active muscle stiffness (e.g., Kubo, 2014). The repeatability of measurements of active muscle stiffness at 250 and 100°·s^−1^ was confirmed in our previous studies (Kubo & Ikebukuro, 2019; Kubo et al., 2018).

### 2.5 Stiffness and hysteresis of tendon

**FIGURE 2:**
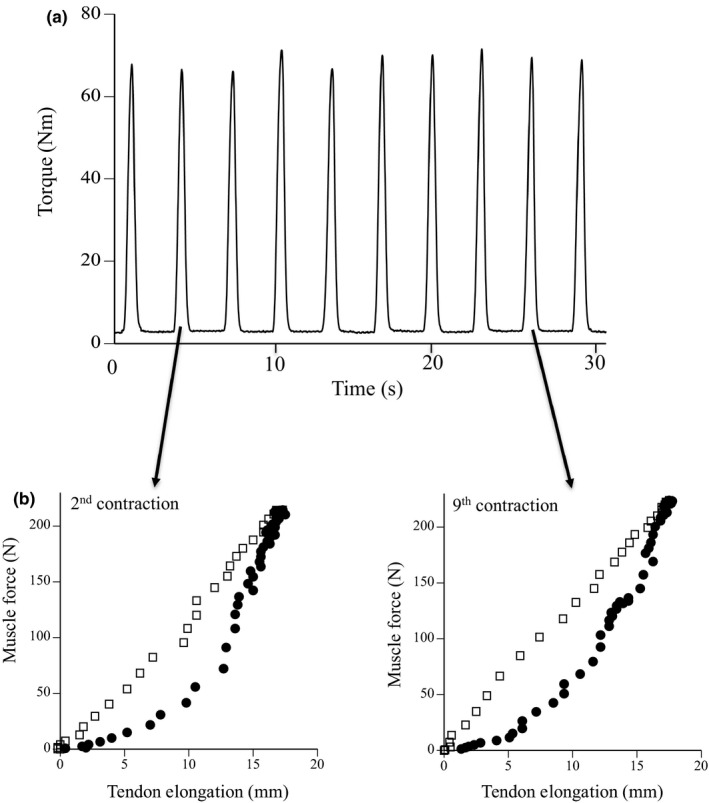
Typical examples of exerted torque during 10 repeated submaximal contractions (a) and muscle force–tendon elongation curves during 2nd and 9th contractions (b)

Stiffness and hysteresis of the tendon during ramp (lower strain rate) and ballistic (higher strain rate) contractions were measured using the procedure described by our previous studies (Kubo & Ikebukuro, 2019; Kubo et al., 2017). Subjects lay prone on the test bench with the foot securely fastened to the footplate of the dynamometer (custom made, Vine) by two straps. The ankle joint was set at 90° with the legs fully extended. In ramp contraction, subjects were instructed to develop a gradually increasing torque from relaxation to MVC within 5 s, followed by a gradual relaxation within 5 s. In ballistic contraction, subjects were instructed to develop isometric torque from relaxation to MVC powerfully and quickly, followed by suddenly relaxation. However, the duration during ascending and descending phases (about 0.5 s) was more or less longer than that in the previous studies (e.g., Penailillo et al., 2015), since subjects were asked to perform ballistic contractions to prioritize to take clear ultrasonic images during measurements (Ishigaki & Kubo, 2020). The measurements for ramp and ballistic contractions were performed twice with a 1‐min rest between tests. In addition, subjects were requested to perform 10 repeated ballistic contractions with a 2‐s gap between repetitions at 50% MVC (Figure 2). During repeated contractions, subjects were encouraged to maintain the target torque displayed on an oscilloscope. The measured values were the means of two contractions (second and ninth contractions). Torque signals were analog‐to‐digital converted as a sampling rate of 1 kHz and analyzed by a computer.

During the measurement of tendon properties, a longitudinal ultrasonic image of MG was recorded on a videotape at 60 Hz. The ultrasonic image, torque, and ankle angle (by an electrical goniometer) were synchronized using a timer. Displacement of the intersection point between fascicle and aponeurosis indicated the lengthening of tendon. However, joint rotation occurred, resulting tendon displacement, even during isometric contraction. According to the procedure previously adopted (e.g., Magnusson et al., 2001), ankle joint angle during the measurement of tendon properties was monitored by an electrical goniometer (Penny and Giles). Additional measurements were performed under passive conditions (rotating the ankle from 90 to 99°) in order to correct the measured tendon elongation. Only tendon elongation values corrected for angular rotation are shown in the present study.

The torque measured by the dynamometer during the measurement of tendon properties was converted to muscle force using the same way as used to measure active muscle stiffness. In this study, the slope of muscle force and elongation of tendon structures above 50% of MVC was defined as tendon stiffness. The elastic energy absorption by the tendon was proposed by calculating the area below the *F*_m_–tendon elongation curve. The ratio of the area within the force–elongation loop to the area beneath the curve during the ascending phase was calculated as tendon hysteresis. The repeatability of measurements of the tendon stiffness and hysteresis measured during ramp and ballistic contractions was confirmed in our previous studies (Kubo & Ikebukuro, 2019; Kubo et al., 2017).

### 2.6 Performance and behavior of fascicles during jumping

Subjects jumped unilaterally using only the ankle joint on the custom‐designed sledge apparatus (AO‐3000K; Applied Office) with following three conditions: no‐countermovement jump (noCMJ), counter‐movement jump (CMJ), and DJ. The vertical reaction force from a force plate (Kistler, 9281B) attached to the footplate of the apparatus was measured during jumping. Retroreflective marks were attached to the lateral epicondyle of the knee, lateral malleolus, and fifth metatarsophalangeal joint. All jump trials were filmed using a digital high‐speed camera at a sampling frequency of 250 Hz (VCC‐H1600C; Digimo).

Prior to the test, subjects performed submaximal jumps to become accustomed to the test procedures. In all tests, they were asked to jump five times for each jumping test (with at least 1 min between tests) as high as possible. In noCMJ, they held the ankle in a maximal dorsiflexed position, and then exerted plantar flexion torque until the toe lifted off the force plate. In CMJ, they held the ankle in a maximal plantar flexed position. Subsequently, they exerted plantar flexion torque to a maximal dorsiflexed position, and suddenly rebounded to exert plantar flexion torque until the toe lifted off the force plate. In DJ, they were dropped from a height of 20 cm from the surface of the force plate by the assistance of an equipment of the sledge apparatus. After landing on the force plate, they voluntarily stopped the falling movement by exerting eccentrically plantar flexion torque, and then started plantar flexion and took off.

The jump height and ankle joint angle were measured by a digitizing software (Image J; NIH). Jump height was assessed as the maximum displacement of the seat of the sledge apparatus from the position at 90° of ankle angle. The averaged values of two trials with the highest and second highest jump heights were adopted. In addition, the difference between the heights of CMJ or DJ and noCMJ, expressed as the percentage of that of noCMJ, was calculated as an index of pre‐stretch augmentation (Kubo, Morimoto, Komuro, Tsunoda, et al., 2007; Kubo, Morimoto, Komuro, Yata, et al., 2007).

A real‐time ultrasonic apparatus (Prosound 7; Hitachi Aloka Medical) was used to obtain longitudinal ultrasonic images of MG during jumping. The scanning probe of the apparatus was secured with adhesive tape at 30% of the lower leg length. Ultrasonic images were recorded on video tape at 60 Hz, synchronized with recordings of a clock timer for subsequent analysis. The fascicle length and pennation angle were defined as the distance between the insertions of the fascicle into the superficial and deep aponeurosis and the angle between the fascicle and deep aponeurosis. Changes in length of the muscle–tendon complex of MG were calculated from the joint angle and lower leg length data using the equations reported by Greive et al. (1978). The changes in tendon length (outer‐tendon and aponeurosis at both proximal and distal ends) were estimated by subtracting the change in the “effective fascicle length” (calculated by multiplying the fascicle length by the cosine of the pennation angle) from the change in muscle–tendon complex length, as described previously (Fukunaga et al., 2001; Kawakami et al., 2002).

In addition, electromyographic (EMG) activity during jumping was sampled at 1 kHz using a wireless EMG telemeter system (BioLog DL‐5500; S&ME). Surface electrodes (DL‐510; S&ME) were placed on the skin over the muscle belly of LG and SOL. The raw data were band‐pass filtered between 10 and 500 Hz. EMG amplitude was rectified and averaged for the following phases during jumping (mEMG); eccentric and concentric phases according to the ankle joint angle. In addition, the mean mEMG of LG and SOL was calculated as the mEMG of the plantar flexor muscles (PF).

### 2.7 Statistical analysis

Values are reported as means ± standard deviation. A two‐way analysis of variance (ANOVA) with repeated measures was used to detect significant effects of time (before and after training) and torque level (%MVC) on changes in torque and fascicle length during the measurement of active muscle stiffness. Regarding other variables, differences in time (before and after training) and group (training and control) were tested using two‐way ANOVA with repeated measures {2 (test times) × 2 (group)}. The *F* ratios for main effects and interactions were considered to be significant. Significant differences among means were detected using the Bonferroni post hoc test. In all ANOVAs, Mauchly's sphericity test was performed to assess the homogeneity of variance. Greenhouse–Geisser correction was applied where the assumption of sphericity was violated. Statistical computations were performed using IBM SPSS Statistics (version 26). Significance was set at *p* < 0.05.

## 3 RESULTS

The muscle thickness significantly increased by 4.7% for MG (*p *= 0.009), 5.4% for LG (*p *= 0.019), and 3.0% for SOL (*p *= 0.021) (Table 1). No significant difference in the relative increase in muscle thickness was found among MG, LG, and SOL (*p *= 0.953). In addition, no significant change in the cross‐sectional area of the tendon was found after training (Table 1).

**TABLE 1:** Morphological properties of muscle and tendon for training and control groups

	Training group		Control group	
	Before	After	Before	After
Muscle thickness of MG (mm)	20.9 (3.1)	21.9 (3.5)**	19.7 (2.1)	20.6 (2.5)
Muscle thickness of LG (mm)	17.3 (2.1)	18.2 (2.5)*	17.9 (2.7)	17.5 (3.0)
Muscle thickness of SOL (mm)	23.2 (4.1)	23.9 (4.6)*	23.5 (3.9)	23.1 (3.6)
Cross‐sectional area (mm^2^)	63.5 (7.2)	64.1 (7.5)	64.0 (6.8)	63.4 (7.0)

Note

Values given are mean (SD).

Abbreviations: LG, lateral gastrocnemius muscle; MG, medial gastrocnemius muscle; SOL, soleus muscle.

Significantly different from before (*p < 0.05, **p < 0.01).

**FIGURE 3:**
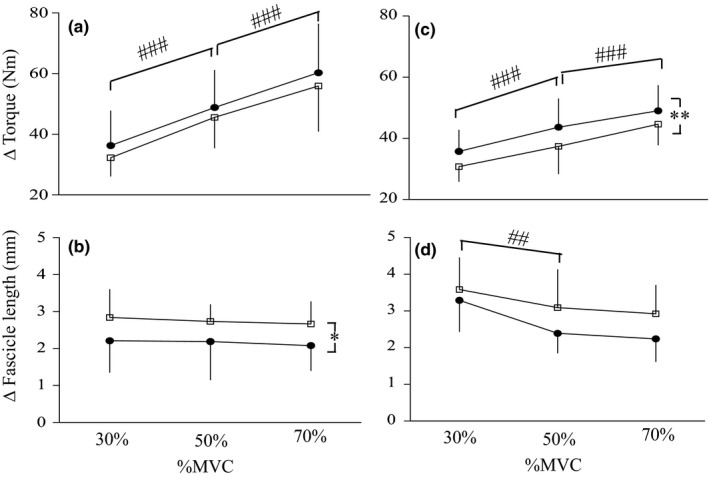
Changes in torque (a, c) and fascicle length (b, d) during fast stretching before (open) and after (closed) training under 250°·s^−1^ (a, b) and 100°·s^−1^ (c, d) conditions. Data are mean ± SD. Significant difference from before training: *p < 0.05, **p < 0.01. Significant difference from the preceding torque level exerted: ## *p *< 0.01, ###*p *< 0.001

Changes in torque and fascicle length under the 250°·s^−1^ condition are shown in Figure 3a,b. Regarding changes in torque, the effect of torque level was significant (*p *< 0.001), whereas the effects of time (*p *= 0.060) and the interaction between time and torque level (*p *= 0.929) were not (Figure 3a). Regarding changes in fascicle length, the effect of time was significant (*p *= 0.011), whereas the effects of torque level (*p *= 0.604) and the interaction between time and torque level (*p *= 0.973) were not (Figure 3b). Active muscle stiffness significantly increased by 62.0% at 30% MVC (*p *= 0.041), 53.0% at 50% MVC (*p *= 0.007), and 50.9% at 70% MVC (*p *= 0.047) after training (Table 2).

**TABLE 2:** Mechanical properties of muscle and tendon for training and control groups

	Training group		Control group	
	Before	After	Before	After
Active muscle stiffness at 250°·s^−1^				
30% MVC	40.3 (15.6)	64.0 (38.0)*	44.9 (16.0)	41.8 (13.5)
50% MVC	55.3 (14.2)	87.4 (41.8)**	58.6 (15.5)	59.2 (16.1)
70% MVC	71.1 (21.9)	107.6 (61.0)*	70.5 (19.6)	74.0 (20.9)
Active muscle stiffness at 100°·s^−1^				
30% MVC	30.2 (10.8)	37.9 (16.5)	31.6 (11.6)	33.9 (12.7)
50% MVC	42.5 (14.4)	62.5 (24.1)*	44.5 (15.2)	45.0 (13.8)
70% MVC	53.4 (18.6)	76.8 (29.7)*	52.6 (20.4)	50.34 (23.2)
Ramp				
Maximal force (N)	342 (89)	356 (104)	333 (97)	341 (88)
Maximal elongation (mm)	18.2 (4.1)	18.2 (4.0)	17.5 (5.2)	18.0 (5.5)
Stiffness (N·mm^−1^)	30.3 (7.0)	27.0 (6.2)	27.9 (7.8)	28.3 (10.9)
Elastic energy (J)	2.6 (1.1)	2.8 (1.5)	2.4 (1.4)	2.5 (1.2)
Hysteresis (%)	16.0 (11.3)	18.9 (11.8)	18.2 (13.0)	17.4 (10.9)
Ballistic				
Maximal force (N)	365 (89)	372 (104)	344 (95)	348 (101)
Maximal elongation (mm)	14.6 (3.2)	16.7 (3.5)*	15.3 (4.1)	15.5 (3.8)
Stiffness (N·mm^−1^)	22.0 (7.1)	21.7 (6.8)	26.9 (7.7)	26.0 (8.2)
Elastic energy (J)	2.8 (1.1)	3.4 (1.1)**	2.6 (1.3)	2.8 (1.6)
Hysteresis (%)	48.4 (4.9)	38.3 (10.9)*	44.7 (9.9)	46.1 (9.5)
Submaximal repeated				
Hysteresis (%)	46.5 (4.9)	35.5 (8.1)**	43.9 (10.5)	44.5 (9.1)

Note

Values given are mean (SD).

Significantly different from before (*p < 0.05, **p < 0.01).

Changes in torque and fascicle length under the 100°·s^−1^ condition are shown in Figure 3c,d. Regarding changes in torque, the effects of torque level (*p *< 0.001) and time (*p *= 0.003) were significant, whereas the effect of the interaction between time and torque level was not (*p *= 0.701) (Figure 3c). Regarding changes in fascicle length, the effect of torque level was significant (*p *< 0.001), whereas the effects of time (*p *= 0.094) and the interaction between time and torque level (*p *= 0.437) were not (Figure 3d). Active muscle stiffness significantly increased by 59.2% at 50% MVC (*p *= 0.027) and 56.3% at 70% MVC (*p *= 0.037), but not at 30% MVC (*p *= 0.183), after training (Table 2).

**FIGURE 4:**
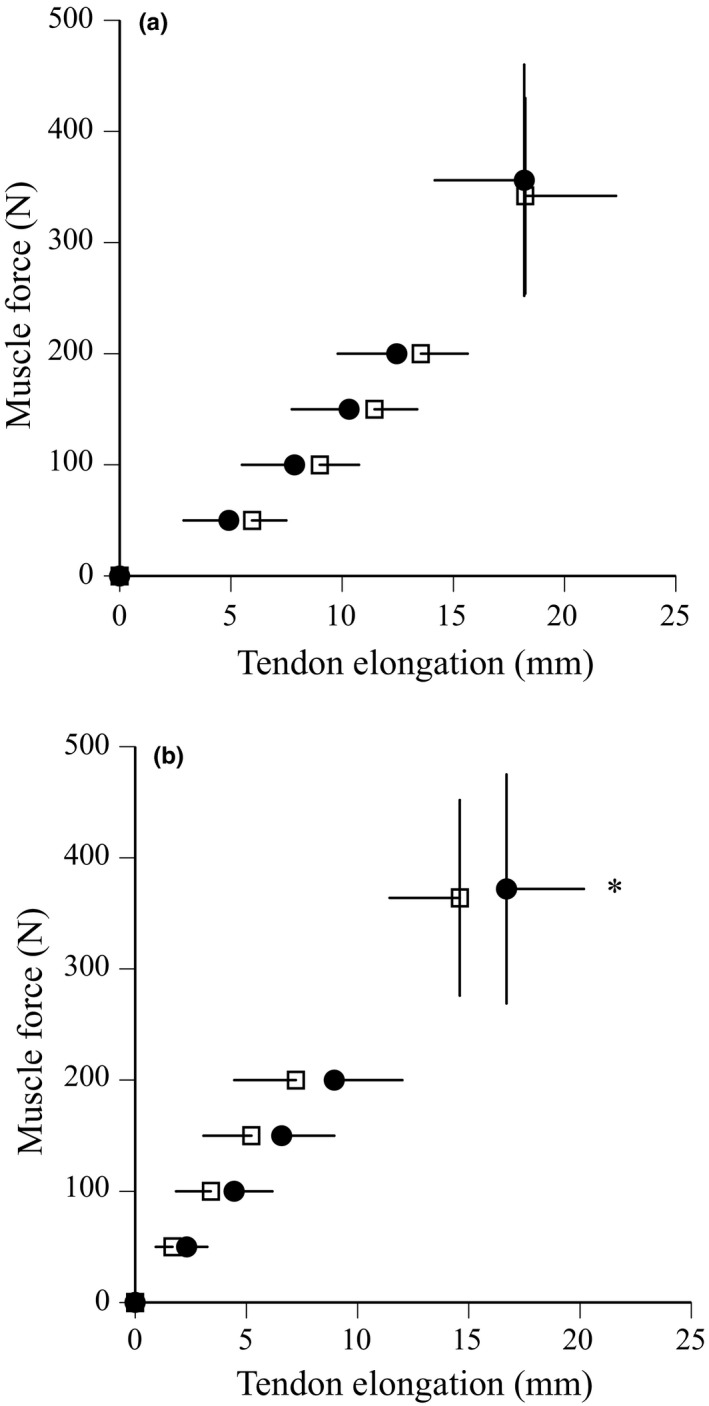
Relationships between muscle force and tendon elongation during ramp (a) and ballistic (b) contractions before (open) and after (closed) training. Data are mean ± SD. Significant difference from before training: *p < 0.05

**FIGURE 5:**
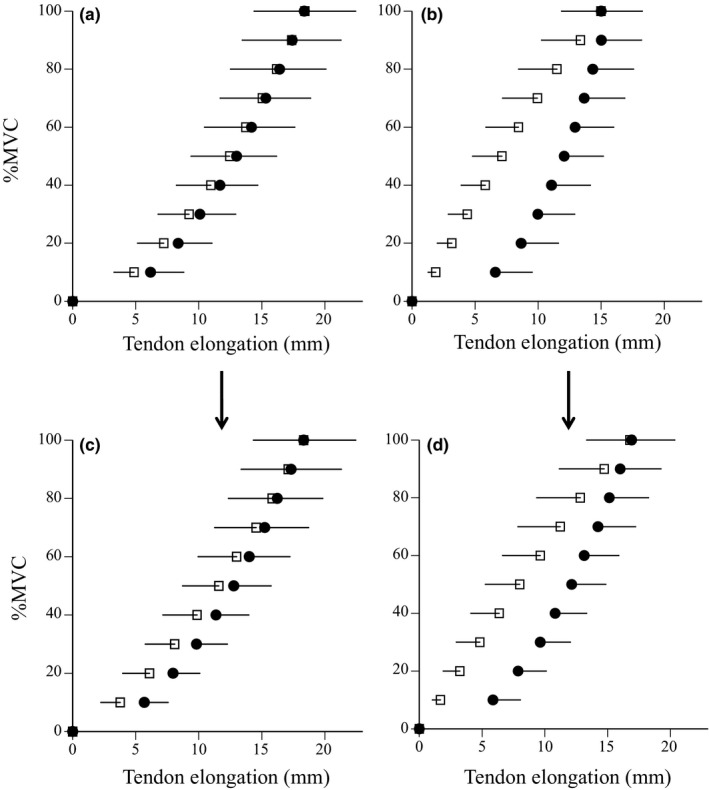
Relationship between %MVC and tendon elongation during ramp (left: a and c) and ballistic (right: b and d) contractions before (upper: a and b) and after (lower: c and d) training. Data are mean ± SD

**FIGURE 6:**
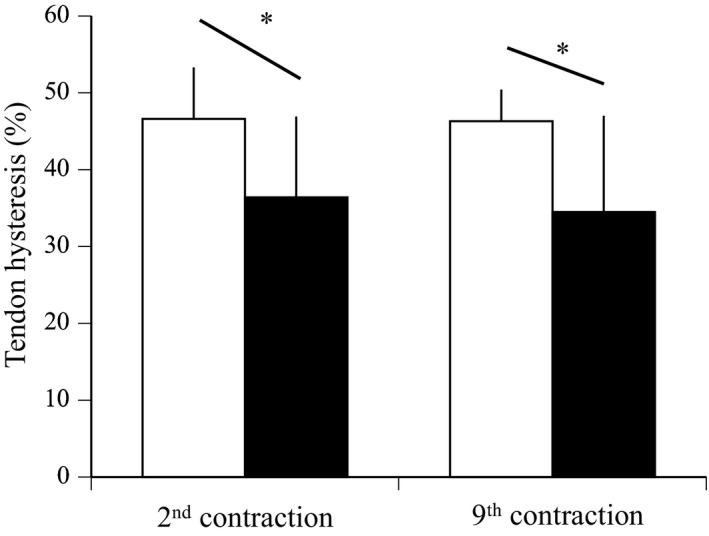
Tendon hysteresis at 2nd and 9th contractions during 10 repeated submaximal contractions. Data are mean ± SD. Significant difference from before training: *p < 0.05

The relationships between estimated muscle force and tendon elongation during ramp and ballistic contractions are shown in Figure 4. In ramp and ballistic contractions, maximal muscle force did not change after training (Table 2). Maximal tendon elongation (*p *= 0.018) and elastic energy (*p *= 0.005) during ballistic contraction significantly increased after training, whereas those during ramp contraction did not (*p *= 0.954 for maximal tendon elongation, *p *= 0.143 for elastic energy) (Table 2). In ramp and ballistic contractions, tendon stiffness did not change after training (Table 2; Figure 4). Tendon hysteresis during ballistic contraction significantly decreased after training (*p *= 0.012), whereas that during ramp contraction did not (*p *= 0.364) (Table 2, Figure 5). In addition, tendon hysteresis during submaximal repeated contractions significantly decreased for 2nd and 9th contractions (Figure 6) and for means of two contractions (*p *= 0.003, Table 2).

**FIGURE 7:**
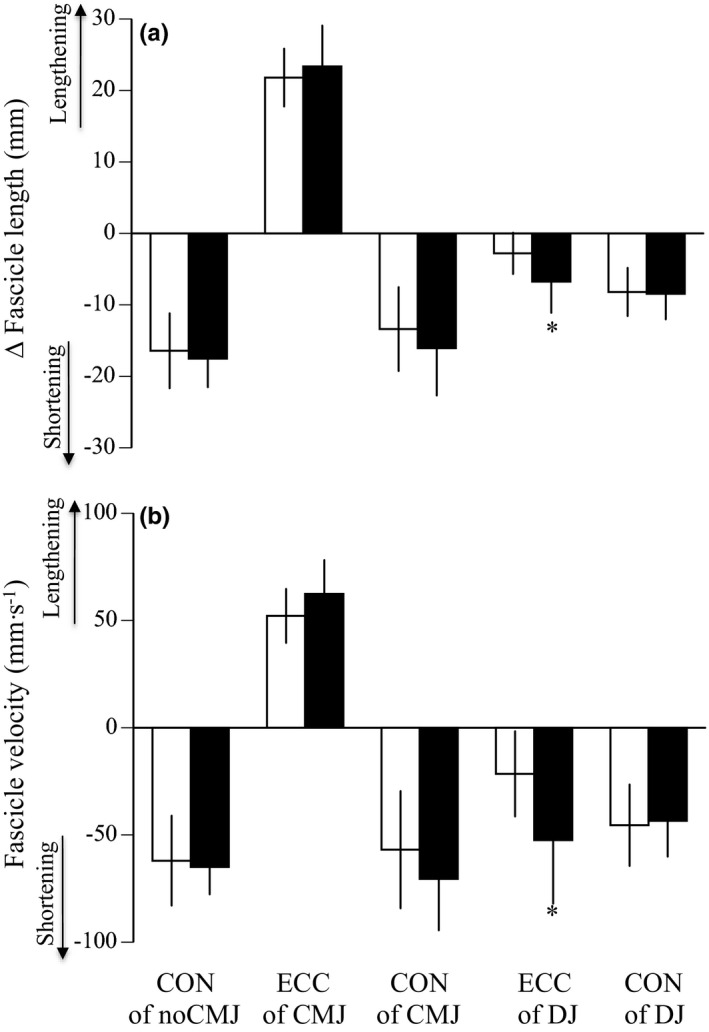
The changes in fascicle length (a) and fascicle velocity (b) during eccentric (except for noCMJ) and concentric phases of noCMJ, CMJ, and DJ before (open) and after (closed) training. CON; concentric phase, ECC; eccentric phase, noCMJ; no counter‐movement jump, CMJ; counter‐movement jump, DJ; drop jump. Data are mean ± SD. Significant difference from before training: *p < 0.05

**FIGURE 8:**
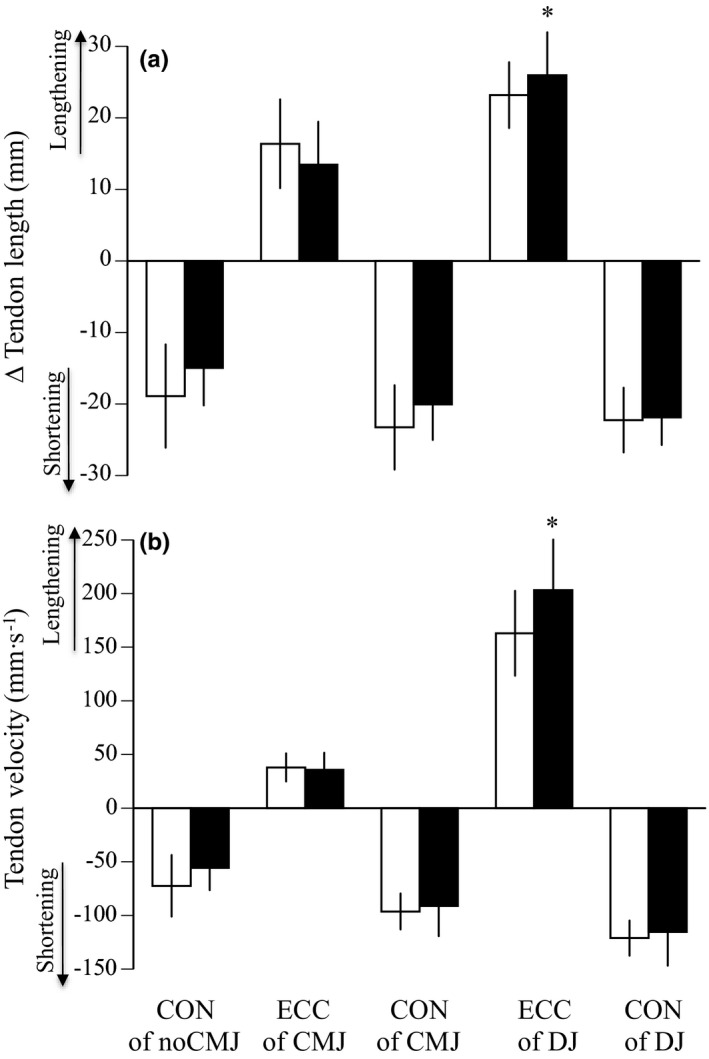
The changes in tendon length (a) and tendon velocity (b) during eccentric (except for noCMJ) and concentric phases of noCMJ, CMJ, and DJ before (open) and after (closed) training. CON; concentric phase, ECC; eccentric phase, noCMJ; no counter‐movement jump, CMJ; counter‐movement jump, DJ; drop jump. Data are mean ± SD. Significant difference from before training: *p < 0.05

Table 3 shows the variables measured during the three kinds of jumping tests before and after training. Jump heights significantly increased by 24.0% for noCMJ (*p *= 0.025), 47.0% for CMJ (*p *< 0.001), and 30.0% for DJ (*p *< 0.001). The pre‐stretch augmentation in CMJ significantly increased after training (*p *< 0.001), whereas that in DJ did not (*p *= 0.378). No significant differences in the ankle angle at the lowest position, angular velocities, and mEMG of PF were found before and after training. The ratio of mEMG value during the eccentric to concentric phase of CMJ significantly increased after plyometric training (*p *= 0.004), although that of DJ did not (*p *= 0.365). For the fascicle, the amount of fascicle shortening (*p *= 0.009) and shortening velocity (*p *= 0.006) during the eccentric phase of DJ significantly increased after training, although those during other phases of the three jump tests did not (Figure 7). For the tendon, the amount of tendon lengthening (*p *= 0.036) and lengthening velocity (*p *= 0.018) during the eccentric phase of DJ significantly increased after training, although those during other phases of the three jump tests did not (Figure 8).

**TABLE 3:** Measured variables during jumping tests for training and control groups

	Training group		Control group	
	Before	After	Before	After
Ankle angle at the lowest position (°)				
noCMJ	−21.0 (7.4)	−24.4 (5.7)	−20.5 (8.1)	−21.4 (6.6)
CMJ	−21.2 (6.9)	−21.7 (5.0)	−21.1 (7.7)	−20.8 (6.9)
DJ	−14.2 (5.5)	−12.2 (5.9)	−13.9 (6.2)	−14.4 (7.1)
Angular velocity during ECC (°·s^−1^)				
CMJ	115.6 (17.9)	126.3 (23.0)	121.2 (20.9)	117.9 (22.2)
DJ	201.0 (31.0)	199.9 (42.9)	193.5 (34.2)	198.3 (39.9)
Angular velocity during CON (°·s^−1^)				
noCMJ	169.7 (36.6)	170.1 (24.8)	175.0 (31.1)	179.3 (25.5)
CMJ	194.4 (27.2)	205.7 (31.4)	195.2 (29.9)	191.0 (35.4)
DJ	214.8 (33.9)	196.3 (35.9)	207.8 (30.6)	202.9 (33.2)
Jump height (cm)				
noCMJ	11.5 (3.4)	13.6 (1.8)*	12.0 (2.1)	12.2 (2.8)
CMJ	14.8 (4.7)	20.6 (4.0)***	15.1 (3.5)	14.9 (3.9)
DJ	16.7 (3.6)	21.4 (3.6)***	16.3 (4.1)	16.6 (4.4)
Pre‐stretch augmentation (%)				
CMJ	28.0 (17.2)	50.6 (19.0)***	26.9 (15.4)	23.0 (17.2)
DJ	47.5 (14.9)	57.9 (24.6)	37.1 (16.6)	36.5 (20.0)
mEMG of PF during ECC (mV·s^−1^)				
CMJ	0.027 (0.009)	0.035 (0.020)	0.031 (0.011)	0.036 (0.018)
DJ	0.066 (0.032)	0.063 (0.030)	0.058 (0.025)	0.061 (0.029)
mEMG of PF during CON (mV·s^−1^)				
noCMJ	0.085 (0.017)	0.097 (0.040)	0.094 (0.033)	0.087 (0.036)
CMJ	0.097 (0.023)	0.100 (0.044)	0.102 (0.025)	0.098 (0.041)
DJ	0.082 (0.022)	0.084 (0.032)	0.089 (0.019)	0.093 (0.037)
Ratio of mEMG during ECC to that during CON				
CMJ	0.281 (0.075)	0.359 (0.079)**	0.299 (0.084)	0.347 (0.090)
DJ	0.848 (0.344)	0.782 (0.253)	0.691 (0.298)	0.656 (0.392)

Note

Values given are mean (SD).

Abbreviations: CON, concentric phase; CMJ, counter‐movement jump; DJ, drop jump; ECC, eccentric phase; mEMG, mean electromyographic activities; noCMJ, no‐counter‐movement jump; PF, plantar flexor muscles.

Significantly different from before (*p < 0.05, **p < 0.01, ***p < 0.001).

For the control group, no significant changes were noted in the measured variables (Tables 1, 2, 3).

## 4 DISCUSSION

The main results of this study were that after 12 weeks of plyometric training: (1) maximal tendon elongation during ballistic contraction and active muscle stiffness with and without the effect of the stretch reflex significantly increased, (2) tendon hysteresis during ballistic and submaximal repeated contractions significantly decreased, and (3) behaviors of fascicles and tendons were optimized without alteration in neuromuscular activities during DJ but not noCMJ or CMJ.

In the present study, maximal tendon elongation during ballistic contraction significantly increased after 12 weeks of plyometric training, whereas that during ramp contraction did not. These results were consistent with our previous finding (Kubo et al., 2017). To date, no consensus has been reached on the plyometric training‐induced changes in elongation and stiffness of tendons (Burgess et al., 2007; Foure et al., 2009, 2010; Hirayama et al., 2017; Kubo et al., 2017; Kubo, Morimoto, Komuro, Yata, et al., 2007; Wu et al., 2010). In most previous studies, however, tendon properties were evaluated during ramp contraction, that is, a markedly lower strain rate. In order to clarify the mechanisms of enhancement of jumping ability due to plyometric training, we need to evaluate tendon elongation during ballistic contractions, that is, similar to the strain rate of the tendon during running and jumping. Werkhausen et al. (2018) reported that fascicle lengthening and velocity of MG during landing significantly reduced after 10 weeks of repeated explosive contraction training (1‐s loading, 5‐s rest) like plyometric training. This result suggests that tendon elongation during ballistic contractions increased after explosive‐type training such as plyometric training. In addition, elastic energy during ballistic contraction, but not ramp contraction, significantly increased after training in this study. Therefore, the present result on the change in tendon extensibility after plyometric training suggests a favorable change to store elastic energy during stretch‐shortening cycle exercises.

Tendon hysteresis measured during ballistic contraction, but not during ramp contraction, significantly decreased after plyometric training. In our previous study, we failed to detect any change in tendon hysteresis under the same conditions (Kubo et al., 2017). This discrepancy between the present and our previous results would be related to the difference in hysteresis values before training (35% in Kubo et al., 2017, 48% in the present study), and thereby trainability for tendon hysteresis may be more favorable in the subjects of the present study than those in our previous study. Furthermore, we determined tendon hysteresis during repeated submaximal contractions in order to simulate tendon hysteresis under the conditions close to normal exercise. We also found that the hysteresis value during submaximal repeated contractions was almost the same as that during ballistic contraction, and both decreased to the same degree after plyometric training. Accordingly, we may conclude that tendon hysteresis under the higher strain rate decreased after plyometric training.

After 12 weeks of plyometric training, active muscle stiffness at 250°·s^−1^ increased by 55% on average at all torque levels. This result agreed with our previous finding (Kubo et al., 2017). In addition, active muscle stiffness at 100°·s^−1^ (including the effect of the stretch reflex) also increased to the same degree (50% on average at all torque levels) as for the 250°·s^−1^ condition (i.e., active muscle stiffness without the stretch reflex) after plyometric training. At the beginning of this study, we expected that the plyometric training‐induced increase in active muscle stiffness including the effect of the stretch reflex would be related to that in jump performance, because performance during stretch‐shortening cycle exercises was affected by the stretch reflex (Horira et al., 1996; Kuitunen et al., 2002). However, there were no significant correlations between changes in active muscle stiffness under the 100°·s^−1^ condition and jump performances in the present study (data not shown). On the other hand, Voigt et al. (1998) reported no change in the short‐latency stretch reflex after 4 weeks of plyometric training. Potach et al. (2009) indicated that long‐latency stretch reflex would change after plyometric training, whereas latency time of the short‐latency stretch reflex did not. In the present study, the duration for calculating active muscle stiffness at 100°·s^−1^ was 110 ms from the onset of the stretch, and thus active muscle stiffness at 100°·s^−1^ may include short‐ and middle‐latency stretch reflexes, but not long‐latency ones. Taking these points into account together with the present results, short (and middle)‐latency stretch reflex did not alter by plyometric training, as described in previous studies (Potach et al., 2009; Voigt et al., 1998).

On the other hand, the change in fascicle length under the 250°·s^−1^ condition significantly decreased after plyometric training, whereas that in torque under the 100°·s^−1^ condition significantly increased. Recently, we also found that active muscle stiffness significantly decreased by 22% under the 250°·s^−1^ condition and 16% under the 100°·s^−1^ condition after repeated hopping exercises (Kubo & Ikebukuro, 2019). In addition, the change in fascicle length under the 250°·s^−1^ condition significantly increased after repeated hopping exercises, whereas that in torque under the 100°·s^−1^ condition significantly decreased (Kubo & Ikebukuro, 2019). Considering these findings, active muscle stiffness under the 250°·s^−1^ condition may depend on the change in fascicle length, whereas that under the 100°·s^−1^ condition may depend on the change in torque. Unfortunately, we have no data allowing discussion of the definite physiological background behind these phenomena.

The noCMJ and CMJ heights significantly increased by 24% and 47%, respectively, and the pre‐stretch augmentation of CMJ significantly increased after plyometric training. Considering these results, an increase in CMJ height would be associated with changes in tendon properties (elongation and hysteresis), since the mechanical properties of tendons affect the pre‐stretch augmentation during stretch‐shortening cycle exercises (Kubo et al., 1999; Kubo, Morimoto, Komuro, Tsunoda, et al., 2007). In the present study, however, behavior of fascicles during CMJ did not change after plyometric training. Regarding muscle activities during CMJ, the ratio of the mEMG value during eccentric to concentric phases significantly increased after plyometric training, although each mEMG value during eccentric and concentric phases did not change (Table 3). This result implied that more elastic energy was stored in the tendons due to an increase in muscle activities of PF during the eccentric phase of CMJ, although changes in behaviors of fascicles and tendons were not found during CMJ. Considering these results, it is likely that the increase in CMJ height due to plyometric training is related to both changes in muscle activation strategies during jumping and the muscle–tendon mechanical properties. This was supported by our previous finding (Kubo, Morimoto, Komuro, Tsunoda, et al., 2007), which indicated that the greater jump height in CMJ compared with noCMJ could be explained by both the tendon elasticity and increased activation level of muscles.

During the eccentric phase of DJ, the degree of fascicle shortening and tendon lengthening significantly increased after plyometric training (Figures 7a and 8a). These results would be associated with the plyometric training‐induced increase in tendon extensibility during ballistic contraction. Unlike CMJ, as described above, the muscle activation strategies did not change during DJ. Hence, it is likely that the training‐induced changes in the muscle–tendon mechanical properties played a more significant role in the enhancement of jump height during DJ. This was supported by our previous finding (Kubo, Morimoto, Komuro, Tsunoda, et al., 2007), which indicated that the greater jump height in DJ compared with noCMJ could be explained by the tendon elasticity, but not by the increased activation level of muscles. On the other hand, Hirayama et al. (2017) reported that the shortening velocity of the Achilles tendon in the first half of the concentric phase during DJ significantly increased after 12 weeks of plyometric training. This result suggests that tendon hysteresis decreased after plyometric training, although they did not show these data on tendon hysteresis. However, a similar result was not obtained in the present study (Figure 8b). The discrepancy between the previous and present results may be related to the method to divide each phase during jumping (four phases in Hirayama et al., 2017, two phases in the present study). Unfortunately, we did not conduct the same procedure as Hirayama et al. (2017), because the criterion of dividing half phases during each eccentric and concentric phase was unclear.

In the present study, we must draw attention to the limitations and assumptions of the methodology followed. First, we estimated the relative contribution of MG within the plantar flexor muscles in terms of the physiological cross‐sectional area from the literature (Fukunaga et al., 1996). At present, it is difficult to quantify the relative contribution of each muscle among synergistic muscles. The present result showed that there were no significant difference in the relative increase in muscle thickness among MG, LG, and SOL. Hence, we may say that the calculation of muscle force based on the assumption as described above was valid to determine the changes in muscle–tendon mechanical properties after plyometric training. Second, we determined the behavior of only the MG fascicles during jumping in order to elucidate the mechanisms underlying improved jumping performance after plyometric training. Previous studies demonstrated that there was no difference in behavior of MG and SOL during isokinetic plantar flexion and ankle bending exercise (Chino et al., 2008; Sakuma et al., 2012), although the triceps surae muscles (MG, LG, and SOL) had different fiber compositions and architectural characteristics. On the other hand, other researchers showed that there were distinct differences in the dynamics of the muscle fascicles and tendons during stretch‐shortening cycle exercises (e.g., Sousa et al., 2007). In a future study, we need to investigate whether the behavior of MG represents that of all triceps surae muscles.

In conclusion, maximal tendon elongation and elastic energy during ballistic contraction and active muscle stiffness with and without the effect of the stretch reflex significantly increased, and tendon hysteresis during ballistic and submaximal repeated contractions significantly decreased after 12 weeks of plyometric training. Furthermore, the behaviors of the fascicles and tendons were optimized without alteration in neuromuscular activities during DJ but not CMJ. These results indicate that increases in the CMJ height were associated with changes in the muscle–tendon properties and muscle activation strategy, whereas the increase in the DJ height could be explained by changes in the muscle–tendon properties, but not by the muscle activation strategy.

## CONFLICT OF INTEREST

No conflict of interest, financial or otherwise, are declared by the authors.

## AUTHOR CONTRIBUTIONS

K.K., T.I., and H.Y. conceived and designed research; K.K. and T.I. performed experiments; K.K. and T.I. analyzed data; K.K. and H.Y. interpreted results of experiments; T.I. and H.Y. prepared figures; K.K. and H.Y. drafted manuscript; K.K., T.I., and H.Y. edited and revised manuscript; K.K., T.I., and H.Y. approved final version of manuscript.
